# Evaluation of In Vitro Serotonin-Induced Electrochemical Fouling Performance of Boron Doped Diamond Microelectrode Using Fast-Scan Cyclic Voltammetry

**DOI:** 10.3390/bios14070352

**Published:** 2024-07-19

**Authors:** Mason L. Perillo, Bhavna Gupta, James R. Siegenthaler, Isabelle E. Christensen, Brandon Kepros, Abu Mitul, Ming Han, Robert Rechenberg, Michael F. Becker, Wen Li, Erin K. Purcell

**Affiliations:** 1 Department of Biomedical Engineering, Institute for Quantitative Health Science and Engineering, East Lansing, MI 48824, USA; perillom@msu.edu (M.L.P.); chris897@msu.edu (I.E.C.).; wenli@msu.edu (W.L.); 2 Neuroscience Program, Michigan State University, East Lansing, MI 48824, USA; guptabh2@msu.edu; 3 Fraunhofer USA Center Midwest, Coatings and Diamond Technologies Division, East Lansing, MI 48824, USA; jsiegenthaler@fraunhofer.org (J.R.S.); bkepros@fraunhofer.org (B.K.); rrechenberg@fraunhofer.org (R.R.); mbecker@fraunhofer.org (M.F.B.); 4 Department of Electrical and Computer Engineering, Michigan State University, East Lansing, MI 48824, USA; mitulabu@msu.edu (A.M.); mhan@egr.msu.edu (M.H.)

**Keywords:** serotonin, fast-scan cyclic voltammetry, boron-doped diamond, sensing, neuroscience, biomedical engineering, dopamine, electrochemical fouling, neurotransmitter detection, microelectrodes

## Abstract

Fast-scan cyclic voltammetry (FSCV) is an electrochemical sensing technique that can be used for neurochemical sensing with high spatiotemporal resolution. Carbon fiber microelectrodes (CFMEs) are traditionally used as FSCV sensors. However, CFMEs are prone to electrochemical fouling caused by oxidative byproducts of repeated serotonin (5-HT) exposure, which makes them less suitable as chronic 5-HT sensors. Our team is developing a boron-doped diamond microelectrode (BDDME) that has previously been shown to be relatively resistant to fouling caused by protein adsorption (biofouling). We sought to determine if this BDDME exhibits resistance to electrochemical fouling, which we explored on electrodes fabricated with either femtosecond laser cutting or physical cleaving. We recorded the oxidation current response after 25 repeated injections of 5-HT in a flow-injection cell and compared the current drop from the first with the last injection. The 5-HT responses were compared with dopamine (DA), a neurochemical that is known to produce minimal fouling oxidative byproducts and has a stable repeated response. Physical cleaving of the BDDME yielded a reduction in fouling due to 5-HT compared with the CFME and the femtosecond laser cut BDDME. However, the femtosecond laser cut BDDME exhibited a large increase in sensitivity over the cleaved BDDME. An extended stability analysis was conducted for all device types following 5-HT fouling tests. This analysis demonstrated an improvement in the long-term stability of boron-doped diamond over CFMEs, as well as a diminishing sensitivity of the laser-cut BDDME over time. This work reports the electrochemical fouling performance of the BDDME when it is repeatedly exposed to DA or 5-HT, which informs the development of a chronic, diamond-based electrochemical sensor for long-term neurotransmitter measurements in vivo.

## 1. Introduction

The central nervous system (CNS) uses chemical messengers called neurotransmitters to transmit information between neurons [1]. Many neurologic and neuropsychiatric disorders cause dysregulations in neurotransmitter function, and the neurological underpinnings of behavior can be uncovered by studying neurotransmitter dynamics [2,3,4,5,6,7,8,9]. Fast-scan cyclic voltammetry (FSCV) is a powerful method to study the real-time release and reuptake of electroactive neurotransmitters. FSCV is an electrochemical method that typically uses a carbon fiber microelectrode (CFME) referenced to an Ag/AgCl electrode to detect redox currents from electroactive analytes by applying a cyclic potential waveform at high scan rates. This method utilizes the magnitude and voltage associated with the redox current peaks to estimate the concentrations and identity of an analyte, respectively [10]. FSCV is particularly useful for the detection of monoamine neurotransmitters, such as catecholamines (dopamine (DA), norepinephrine, and epinephrine) as well as indolamines (serotonin (5-HT) and histamine) [11,12,13,14,15,16].

Monoamine neurotransmitters can produce reaction products following oxidation. In particular, 5-HT induces the formation of polymerizing oxidative byproducts that have a higher adsorption affinity to the CFME than 5-HT itself, disrupting electron transfer and subsequent signal detection [15,17,18,19]. This phenomenon, known as “electrochemical fouling”, is separate from “biofouling”, which arises from the adsorption of other biological interferents to the electrode surface, such as blood, damaged tissue, etc. [10,20,21,22]. Both types of fouling can interfere with in vivo FSCV signal detection, especially in the chronic setting where exposure to fouling agents may be prolonged. A variety of strategies have been explored to combat electrode fouling. Generally, these strategies treat both types of fouling as one phenomenon, but some groups, such as Weese et al., have shown that the two types of fouling may have unique requirements to mitigate them best. They found electrochemical fouling resistance can be achieved with electrodes that have a pristine surface, whereas biofouling resistance is achieved with functionalization with hydrophilic groups, indicating a different mechanism between the two fouling types [17]. Generally, to resist both types of fouling, researchers have explored new carbon materials, coatings, and alternative waveform parameters. Alternative carbon materials such as carbon nanotubes (CNTs) [17,23,24], nanodiamonds (NDs) [24,25,26], and boron-doped diamond (BDD) [21,27,28,29,30,31,32,33,34,35,36] have shown promise in reducing fouling by leveraging their unique surface characteristics, (e.g., polarity, defect sites, and carbon-bonding structures). Various coatings have been developed to reduce fouling, including Nafion, base-hydrolyzed cellulose acetate, and fibronectin [20]. Several studies have explored adjusting waveform parameters to mitigate both biofouling and electrochemical fouling through various mechanisms [14,21,37,38,39,40]. Oxidative etching driven by increased switching potentials (>1.1 V) will constantly refresh the CFME surface, which can remove fouling agents of both types but will degrade the electrode over time [41]. The “Jackson” N-shaped waveform was designed to outrun and reject adsorption of the oxidative byproducts of 5-HT by altering the scan rate and potentials of the waveform to avoid the adsorption of byproducts while maintaining sensitivity and selectivity for 5-HT [15]. Similarly, our recent report indicated that when using the Jackson waveform, the effects of biofouling were less pronounced on boron-doped diamond microelectrodes (BDDMEs) in comparison to CFMEs [21].

**Figure 1:**
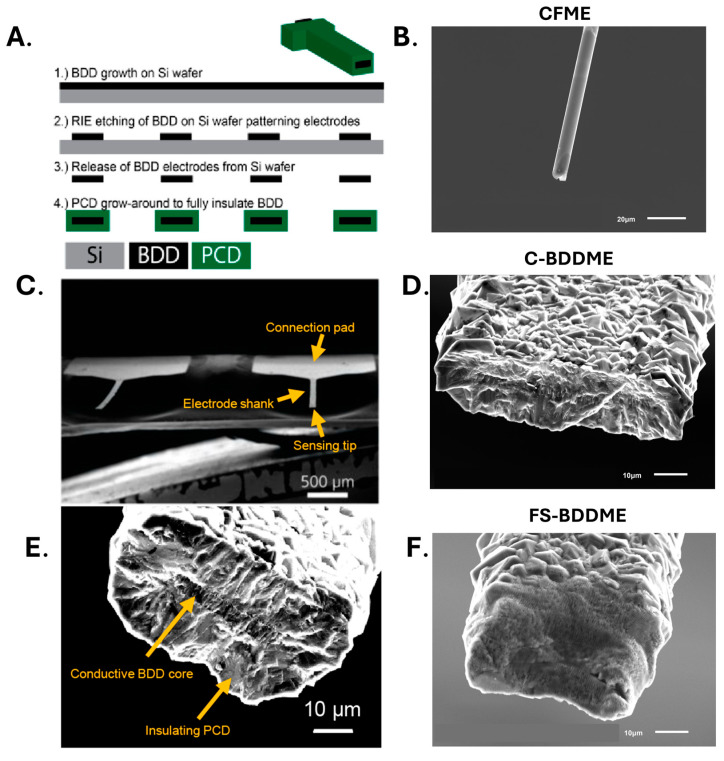
BDDME fabrication and representative devices. (**A**) Material and geometry pattern for silicon (Si) wafer-based chemical vapor deposition. Boron-doped diamond (BDD) is grown on a Si wafer, and then reactive ion etching (RIE) is used to pattern the BDD. The BDD probes are released from the Si wafer, and the insulating polycrystalline diamond (PCD) grows around the BDD shanks. (**B**) Representative CFME tip at 900× magnification. (**C**) Scanning electron microscope (SEM) image of the BDDME shank, integrated connection pad, and the sensing tip. (**D**) C-BDDME tip at 1500× magnification. (**E**) SEM image of the sensing tip of a C-BDDME. The conductive BDD core is exposed from the PCD during fabrication by cleaving the end of the shank with a knife or by laser-cutting the shank with a femtosecond laser. (**F**) FS-BDDME tip at 1500× magnification. The left column of this figure is adapted from [21].

Our group is developing a freestanding, all-diamond, batch-fabricated BDDME (Figure 1) as an alternative to the CFME to mitigate fouling while reducing etching potentially [21,28,42]. The device is batch-fabricated using a wafer-scalable process that can produce hundreds of identical microelectrodes simultaneously without the need for cumbersome manual assembly. The BDDME is a fiber-style electrode grown on silicon wafers using a combination of microwave chemical vapor deposition (CVD) to grow the BDD and hot-filament CVD to insulate the conductive core with polycrystalline diamond (PCD). This approach enables readily customizable geometries to be fabricated to meet the needs of various sensing configurations. Generally, BDD also provides several other purported benefits when used for electrochemical sensing, such as a wider working potential window, stable background currents, and improved resistance to mass loss due to oxidative etching [27,31,42,43]. Many of these desirable characteristics arise from diamond’s *sp*^3^ hybridized carbon bonding structure, which can create a tradeoff in sensitivity from the *sp*^2^ hybridized bonding of other carbon materials [44].

Our previous work with our BDDME includes an initial characterization [42] and explorations into FSCV waveform development and biofouling performance [21,39]. Given our confirmation that the BDDME resists biofouling relative to the CFME, we wanted to test the electrochemical fouling performance of our electrodes. Concurrently, while exploring fabrication techniques to improve the sensitivity and consistency of our devices, we chose to characterize femtosecond laser cutting as an alternative method to expose the BDD electrode site at the fiber tip. In previous work, we completed this fabrication step by physically cleaving the tip of the electrode with a scalpel. Femtosecond lasers, often used in ophthalmic surgery, offer an extremely precise, focused laser, which is known for its reduced propensity to damage unintended areas surrounding the focal point through minimized thermal flux [45]. We are also exploring the femtosecond laser for an “on-wafer” fabrication step, automating a portion of fabrication normally performed by hand utilizing motorized translation stages.

This study compared 5-HT electrochemical fouling performance between the CFME, the physically cleaved BDDME (C-BDDME), and the femtosecond laser-cut BDDME (FS-BDDME). After initially testing differences in sensitivity, the electrochemical fouling was explored for both DA and 5-HT with similar methods to previous work [14,17] using both a moderate concentration of 5-HT (5 µM) and a large concentration (50 µM). Additionally, we provide an analysis of these devices’ long-term stabilities to explore further the suitability of BDDME as a chronic FSCV sensor, where long-term stability is especially important. We found the following: (1) The C-BDDME resists electrochemical fouling from 5-HT compared with the CFME, and the FS-BDDME at both 5 and 50 µM 5-HT, (2) FS-BDDMEs have sensitivities comparable to CFMEs, but do not maintain the fouling resistance and stability of C-BDDMEs, (3) the C-BDDME has a stable background when exposed to the DA FSCV waveform at 60 Hz for 24 h whereas the CMFE and FS-BDDME both experience changes. We intend this work to be additive to efforts toward viable, chronic neurochemical sensors.

## 2. Materials and Methods

### 2.1. Chemicals

All chemicals were purchased from Sigma-Aldrich, Inc. (St. Louis, MO, USA) and Fisher Scientific International, Inc. (Hampton, NH, USA). Stock solutions of 1 mM DA and 1 mM 5-HT were prepared in 1 mM perchloric acid to prevent degradation. Dilutions of DA and 5-HT were prepared in “Tris” artificial cerebrospinal fluid (aCSF) (pH 7.4; 25 mM Trizma Buffer, 126 mM NaCl, 2.5 mM KCl, 1.2 mM NaH_2_PO_4_, 2.4 mM CaCl_2_, and 1.2 mM MgCl_2_) [22]. Solutions of 1 mM ferrocene carboxylic acid (FcCOOH), an electroactive compound with a symmetrical, well-defined redox response, were prepared in Tris aCSF and used to test for optimal placement of microelectrodes in the flow injection setup prior to measurements. All solutions were prepared with ultrapure water: 18.2 MΩ.cm, TOC < 5 ppb (Barnstead™ GenPure™ xCAD Plus Ultrapure Water Purification System, Thermo Scientific, Waltham, MA, USA).

### 2.2. Carbon Fiber Microelectrode (CFME) Fabrication

CFMEs were constructed similarly to previously reported methods [21]. Briefly, individual 7.4 μm diameter, unsized AS4 carbon fibers (Hexel, Stamford, CT, USA) were aspirated into glass capillaries (World Precision Instruments, Sarasota, FL, USA) using a vacuum pump. The capillaries were pulled with a vertical micropipette puller (Stoelting Co., Wooddale, IL, USA). Electrical connections were made by coating 32 AWG silver-plated wire with PELCO conductive carbon-based glue (Ted Pella, Inc., Redding, CA, USA) and inserting the wire into the open end of the capillary. After 24 h, the wire was epoxied in place. The carbon fibers were cut to an approximate 100–150 μm exposed length measured from the glass seal. All CFMEs were allowed to stabilize for 20–30 min using FSCV by applying the standard cyclic waveform of −0.4 V to 1.3 V at 400 V s^−1^, 60 Hz frequency in Tris aCSF, and then allowed to finish stabilizing for 10 min with a reduced frequency of 10 Hz before being used for data collection.

### 2.3. Boron Doped Diamond Fabrication

The all-diamond boron-doped microelectrodes were fabricated using a similar method to our previously published fabrication pathways [21]. Briefly, BDD was grown on a 4″ diameter, 500 µm thick single-side polished silicon wafer using a 915 MHz microwave chemical vapor deposition reactor. Diamond growth synthesis conditions include a 9 kW microwave power with a 900 °C stage temperature and a chamber pressure of 60 Torr with a gas chemistry of 2% methane. During growth, diborane was added to the diamond grown at a B/C ratio of 37,500 ppm to ensure high conductivity. After BDD growth, titanium and copper (Ti: 10 nm/Cu: 500 nm) were deposited using electron-beam evaporation to structure the BDD. Photolithography was used to pattern the metal hard mask (ABM-USA, Inc., Jan Jose, CA, USA) with wet chemical etching, and the diamond was structured using reactive ion etching. The diamond electrodes were then released from the silicon wafer using an HNA etchant with an HF:HNO_3_:CH_3_COOH composition of 5:11:6 and fully insulated with polycrystalline microcrystalline diamond using hot filament chemical vapor deposition (HF-CVD), encasing the microelectrodes in >10 µm intrinsic diamond. Microcrystalline diamond was grown using a base pressure of 35 Torr and 2% methane. After deposition, both ends of the electrodes were physically cleaved to expose the BDD core on both the tip and connection pad, and the electrical connection was made using conductive carbon glue (Ted Pella, Inc., Redding, CA, USA). Electroactive areas for the diamond cores ranged from 100 to 200 µm^2^ based on a 50 µm and 70 µm wide lithography pattern and a BDD growth thickness of ~4 µm.

Femtosecond laser cutting of the tip was performed using a similar setup as our previously fabricated carbon fiber microelectrodes [46]. Briefly, an Astrella-USP-1 K (Coherent Corp., Santa Clara, CA, USA) 800 nm, 1 kHz, 5 W system with a lab-assembled 3-axis stage was used to slice the tips of the microelectrodes [47]. Laser power was attenuated to 400 mW, and the diamond was cleaved by adjusting the *y*-axis rapidly by hand, making 3–4 passes across the electrode tip.

### 2.4. Fast-Scan Cyclic Voltammetry (FSCV)

A two-electrode setup (a working electrode versus a combined quasi-Ag/AgCl reference/counter electrode) was utilized in a custom flow injection cell for FSCV experiments. A custom potentiostat with a variable gain head stage (50 nA/V, 100 nA/V, 200 nA/V, 500 nA/V, and 1 μA/V) was connected to the electrodes for experimentation. Data were collected using a NI-6363 data acquisition card and High-Definition Cyclic Voltammetry (HDCV) software (Version 4, Department of Chemistry, University of North Carolina, Chapel Hill, NC, USA) [48]. These data were filtered using a Bessel 4th-order lowpass filter at 2000 Hz. For all experiments, the flow injection system used a TTL voltage-controlled source to switch a six-way HPLC valve to introduce a bolus of test analyte. A flow rate of 750 µL min^−1^ was used to deliver Tris aCSF buffer by an NE-1000 syringe pump (New Era Pump Systems, Inc., Farmingdale, NY, USA). All experiments were performed in a custom flow injection cell except for the 24-h stability tests. The “standard” waveform (−0.4 V to 1.3 V to −0.4 V at 400 Vs^−1^) was used for all experimentation applied at 10 Hz except for 24-h testing, where it was applied at 60 Hz. All data points are the average of 3 consecutive oxidation peaks unless otherwise noted. All analyte injections are captured in a 30 s window, with the flow-injection valve opening at 5 s and closing at 15 s. Data were subtracted from the background to remove the non-faradaic current component.

Electrodes were calibrated with 5-HT (CFME and FS-BDDME: 0.025–1 µM, C-BDDME: 1–100 µM). A simple linear regression (Microsoft Excel LINEST function) was used to find the slope (reported as sensitivity) and linearity and to calculate the limit of detection (LOD) of different device types by dividing the Y-variance by the slope of the best-fit line using the equation:

(1) $$LOD=\frac{3.3\sigma }{m}$$

where σ is the standard deviation of the y-intercept of the best-fit response, and m is the slope of the calibration curve. This method is described in [49] the “Validation of Analytical Procedures: Text and Methodology (ICH Q2(R1))”. Smaller concentration ranges than the entire working ranges were used to maximize linearity (CFME and FS-BDDME: 0.025–0.2 µM 5-HT, C-BDDME: 2–20 µM 5-HT) [50].

### 2.5. Electrochemical Fouling Protocol

Serotonin fouling was carried out similarly to work published by Weese et al., in 2019 [17]. The working electrode was first exposed to 25 sequential injections (single data points) of 5 µM DA as a non-fouling control, followed by 25 injections of 5 µM 5-HT. New 20 mL vials of analyte dilutions were prepared for every 5 injections to avoid degradation. The injection syringe was refilled as quickly as possible every 5 injections to avoid excessive time in between sets of 5 injections so that self-cleaning is minimized. Due to the signal of C-BDDME in response to 5 µM 5-HT being on the same order as normal system noise (~1 nA), these experiments were repeated with 50 µM DA and 5-HT to generate larger signals with minimized noise interference. Current values were reported as a percentage of the first injection’s peak oxidation current. Electrodes were immediately removed after the 25th 5-HT injection to attempt to preserve visible fouling agents during imaging.

### 2.6. Electrode Stability Analysis

To assess the response repeatability of the devices, 5 µM 5-HT boluses were injected every 10 min for 120 min, leaving the electrode in place with the waveform applied in between injections to allow for surface renewal [41]. To assess longer-term stability, electrodes were placed in a small beaker of Tris aCSF for 24 h with the standard waveform applied at 60 Hz to simulate six days of constant waveform application at 10 Hz. The electrochemical background was collected once at the beginning and once at the end of the experiment.

### 2.7. SEM Imaging

Scanning electron microscopy (SEM) images were collected using a JSM-6610LV SEM (JEOL Ltd., Tokyo, Japan) in the Michigan State University Center for Advanced Microscopy.

### 2.8. Statistics

Raw data were extracted to Excel (Microsoft Inc., Redmond, WA, USA) from the HDCV Version 4 software, and analyses of the limits of detection (LODs), standard *t*-tests, and linear mixed model ANOVA were performed using Excel (Microsoft Inc., Redmond, WA, USA), GraphPad Prism 10 (GraphPad Software Inc., La Jolla, CA, USA), and SPSS (IBM, Armonk, NY, USA), respectively. Statistical significance was defined at the *p* < 0.05 level. All error bars are derived from the standard error of the mean. Figures were produced using GraphPad Prism 10 and Microsoft Office.

### 2.9. Hot-Acid Boiling

BDDMEs (C-BDDMEs and FS-BDDMEs) were removed from their attached PCBs by soaking in methanol for several minutes. The detached BDDMEs were then added to a 500 mL beaker. A total volume of 15 mL of acids was then added to the beaker. Following this, 5 mL of $H_{2}SO_{4}$, 5 mL of $HNO_{3}$, and 5 mL of ${HClO}_{4}$ were pipetted into the 500 mL beaker, ensuring the BDDMEs were completely submerged. $H_{2}SO_{4}$ was added to the beaker first, and then the oxidizers were added subsequently onto the inner sides of the beaker to prevent the exothermic reaction from overheating. A watch glass was placed on top of the beaker and slowly heated to around 230 °C over the course of 30 min. After 30 min, the solution was allowed to cool and then slowly diluted with deionized water. The BDDMEs were then removed from the diluted acid with a small paint brush and reattached to PCBs with conductive carbon glue (Ted Pella, Inc., Redding, CA, USA). FSCV backgrounds were collected before and after acid boiling.

## 3. Results

### 3.1. Serotonin Response

**Figure 2:**
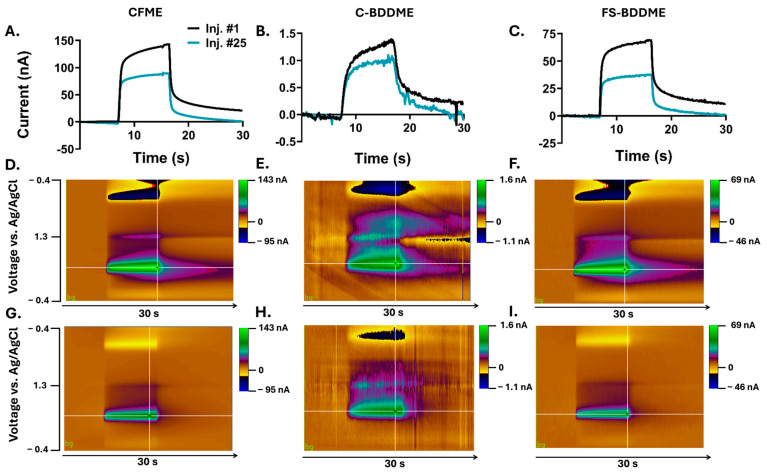
Representative current versus time ((**I**) vs. t) traces and 3D color plots for the CMFE, CBDDME, and FS-BDDME with 5-HT. (**A**–**C**) show the I vs. t plot for a representative 1st and 25th 5-HT injection of 5-HT. (**D**–**F**) depict color plots for the 1st 5-HT response, and (**G**–**I**) show color plots for the 25th.

The compound 5-HT is known to exhibit an attenuated response using FSCV with repeated exposure [15,17,19]. Figure 2A–C depicts the oxidation-peak reduction during a 30 s recording window between the 1st and 25th injection of 5 µM 5-HT across the three device types tested. All three devices show marked reductions in peak current by the 25th injection, with the C-BDDME showing the lowest percent reduction (Figure 2B), the CFME with the second smallest (Figure 2A), and the FS-BDDME with the largest reduction (Figure 2C). Notably, the current magnitudes are highly variable between the three devices. The CFME shown here has a peak current of about 150 nA, the C-BDDME has a peak current of approximately 1.5 nA, and laser cutting brings the peak current magnitude of the BDDME to approximately 75 nA.

### 3.2. Electrode Calibrations

**Figure 3:**
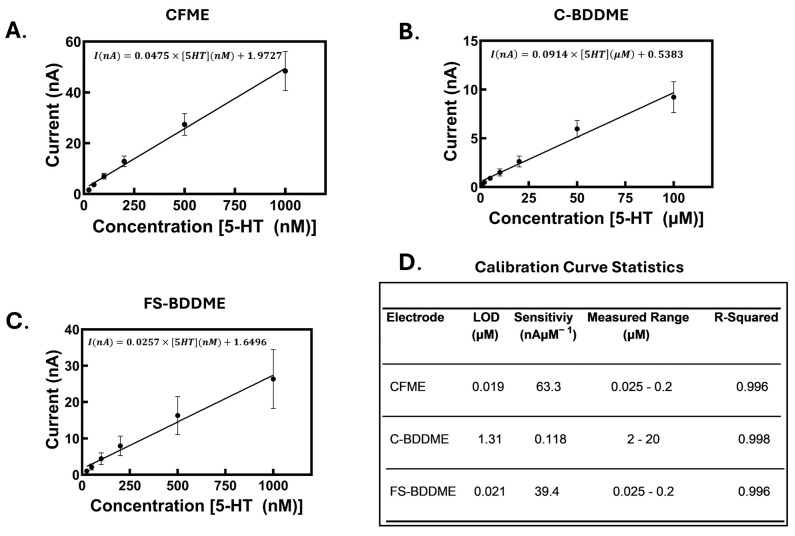
Calibration curves. All plots include the linear regressions for the entire calibrated ranges and their corresponding line equations. (**A**) CFME calibration curve with a slope of 47.5 nAµM^−1^ with a concentration range of 25–1000 nM 5-HT (*n* = 6). (**B**) C-BDDME calibration curve with a slope of 0.0914 nAµM^−1^ with the concentration range of 1–100 µM 5-HT (*n* = 4–5). (**C**) FS-BDDME calibration curve with a slope of 0.0914 nAµM^−1^ with the concentration range of 1–100 µM 5-HT (*n* = 4). (**D**) Calculations performed using the four calibrated data points with the highest linearity (CFME and FS-BDDME: 0.025–0.2 µM 5-HT, C-BDDME: 2–20 µM 5-HT).

Electrodes were calibrated using increasing concentrations of 5-HT to characterize their individual responses. Figure 3 shows the individual calibration curves with their linear regression lines and equations. Due to sensitivity differences between the devices, the concentration ranges of 0.025–1 µM for CFMEs and FS-BDDMEs and 1–100 µM for C-BDDMEs were chosen to reflect their working ranges. All three device types exhibited a highly linear concentration response, with R^2^ values of 0.996 for the CFME (Figure 3A), 0.998 for the C-BDDME (Figure 3B), and 0.996 for the FS-BDDME (Figure 3C). The LODs are as follows: 0.019 µM for the CFME, 1.31 µM for the C-BDDME, and 0.021 µM for the FS-BDDME calculated using Equation (1). The three device types have variable sensitivities. The CFME has a sensitivity of 63.3 nAµM^−1^, and the C-BDDME has a much lower sensitivity of 0.118 nAµM^−1^, likely as a result of its much smaller electroactive surface area and less adsorption, higher sp^3^/sp^2^ hybridized carbon ratio [21,27,28], and the FS-BDDME, functionalized by laser cutting, exhibits a sensitivity much closer to the CFME of 39.4 nAµM^−1^.

### 3.3. Electrochemical Fouling with 5 µM DA and 5-HT

**Figure 4:**
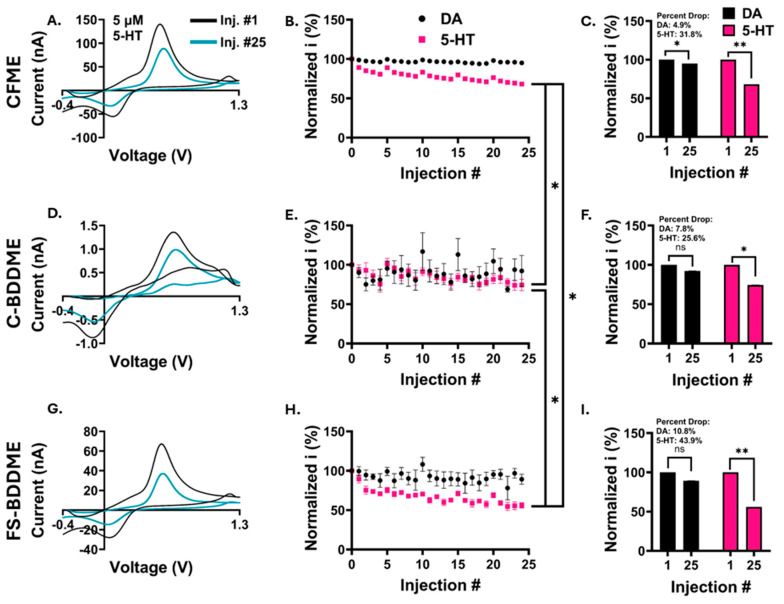
Electrochemical fouling results for 5 µM 5-HT and DA. Current is represented as a percentage of the oxidation peak of the first injection. (**A**,**D**,**G**) Representative (*n* = 1) changes in FSCV voltammograms from the 1st (black) to the 25th (pink) 5-HT bolus injection. (**B**,**E**,**H**) 25 consecutive oxidation peak currents from 5 µM DA (black) and 5 µM 5-HT (pink) (*n* = 6, 4, and 5 from top to bottom). The 5-HT fouling trajectories were significantly different between all three electrode types (*p* < 0.001, linear mixed model ANOVA with a Bonferroni post hoc test, *). (**C**,**F**,**I**) Percent changes from the 1st to the 25th injection for DA (black) and 5-HT (pink). The change in 5-HT peak current from the 1st to 25th injection was also significant for each device type using two-tailed, paired *t*-tests (CFME, *p* < 0.001, **), (C-BDDME, *p* < 0.05, *), and (FS-BDDME, *p* < 0.001, **). The CFME also showed a significant decrease in DA response, although it had the lowest percentage drop of 4.9 ± 1.5% (*t*-test, two-tailed, paired, *p* < 0.05, *).

For all three electrode types, repeated injections showed minimal reductions in DA peak-oxidation currents by the 25th injection and marked reductions in the 5-HT peak-oxidation currents (Figure S3 and Figure 4A,D,G). Slight increases in current following each fifth injection result from solution exchange (see Section 2.5). The CFMEs had an average 31.8 ± 2.4% drop from the first to the last injection (Figure 4B,C, *n* = 6), the C-BDDMEs exhibited a 25.6 ± 7.3% reduction (Figure 4E,F, *n* = 4), and the FS-BDDMEs had a 43.9 ± 3.4% drop (Figure 4H,I, *n* = 5). When comparing responses between device types, the 5-HT injection response curves were all significantly different from each other, with the FS-BDDMEs fouling the most, CFMEs second, and the C-BDDMEs fouling the least (*p* < 0.001, linear mixed model ANOVA with Bonferroni post hoc test). The individual 5-HT percent drops from the 1st to 25th injections were found to be significant for all device types. The individual DA percent drop reached statistical significance only on the CFME. In Figure 4E, it can be observed that the C-BDDME data are more variable than data collected on the other two device types, particularly with DA. This is likely due to the response amplitude being of the same order as system noise [51,52]. While these 5-HT data are clear enough to determine a trend, we chose to repeat these experiments with 50 µM concentrations to better reveal electrochemical fouling responses for the C-BDDME.

### 3.4. Electrochemical Fouling with 50 µM DA and 5-HT

**Figure 5:**
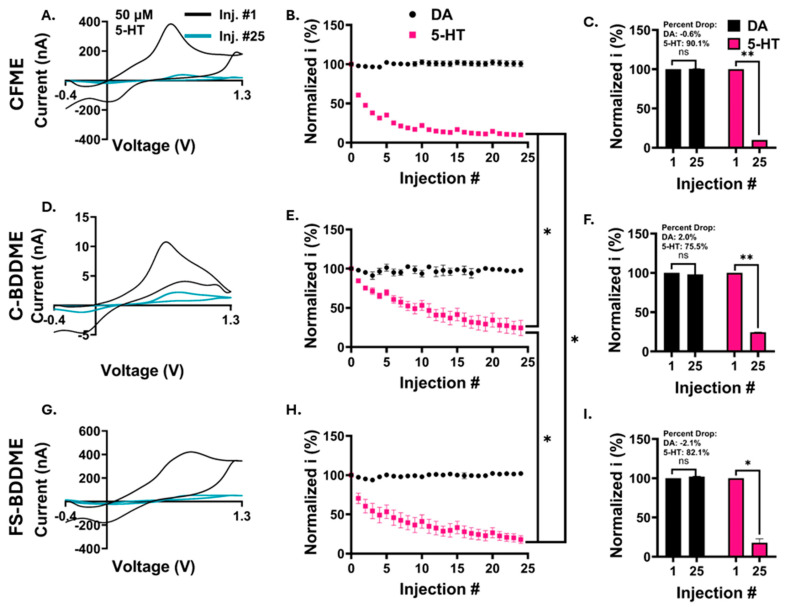
Electrochemical fouling results for 50 µM DA and 5-HT. (**A**,**D**,**G**) Representative (*n* = 1) changes in FSCV voltammograms from the 1st (black) to the 25th (pink) 5-HT bolus injection. (**B**,**E**,**H**) 25 consecutive oxidation peak currents from 50 µM DA (black) and 50 µM 5-HT (pink) (*n* = 4, 4, and 2 from top to bottom). The 5-HT fouling trajectories were significantly different between all three electrode types (*p* < 0.001, linear mixed model ANOVA with a Bonferroni post hoc test, *). (**C**,**F**,**I**) Percent changes from the 1st to the 25th injection for DA (black) and 5-HT (pink). The change in serotonin peak current from the 1st to 25th injection was also significant for each device type using two-tailed, paired *t*-tests (CFME, *p* < 0.001, **), (C-BDDME, *p* < 0.001, **), and (FS-BDDME, *p* < 0.05, *).

As expected, the C-BDDME displayed a less variable response to 50 µM neurotransmitter concentrations than to 5 µM (Figure 4E and Figure 5E), allowing a fouling effect to be revealed with 5-HT. All three device types exhibited exacerbated 5-HT fouling compared with those with lower concentrations. The 5-HT FSCV voltammograms (Figure 5A,D,G) show an almost complete absence of the redox peaks by the 25th injection relative to the 1st, unlike the stable DA FSCV voltammograms (Figure S3). The CFME average 5-HT peak-oxidation current drops from 253 nA to 25.9 nA, the C-BDDME drops from 6.4 nA to 1.3 nA, and the FS-BDDME drops from 224 nA to 30.9 nA. Additionally, the rate of decay in the oxidative peak current is much more pronounced for all devices, particularly with the CFME. The CFMEs had an average 90.1 ± 1.1% peak 5-HT oxidation drop (Figure 5B,C, *n* = 4), the C-BDDMEs reduced by 75.5 ± 9.7% (Figure 5E,F, *n* = 4), and the FS-BDDMEs had an 82.1 ± 4.9% drop (Figure 5H,I, *n* = 2). Similarly to the 5 µM evaluation, there were significant differences in the serotonin trajectories between the three different devices (*p* < 0.001, linear mixed model ANOVA with Bonferroni post hoc test). As for the 5 µM 5-HT test, the FS-BDDMEs had the largest peak oxidation current percent drop relative to the first injection, followed by CFMEs and the C-BDDMEs. The individual 5-HT percent drops from the first to last injections were all found to be significant (*p* < 0.05, paired, two-tailed *t*-test). No statistically significant effects were observed with DA results for 50 µM tests.

### 3.5. Electrode Stability Analysis

**Figure 6:**
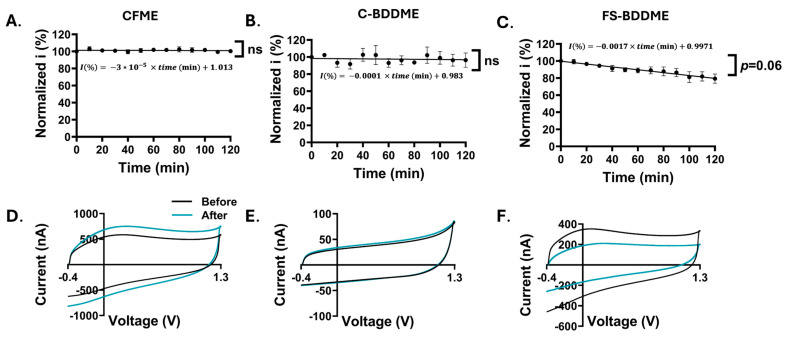
Electrochemical stability analysis with response repeatability and etching stability. Oxidation peak currents are normalized to the 0-min values. (**A**–**C**) show 5-HT response repeatability over a two-hour recording period with 5 µM 5-HT injections every 10 min. Both the CFME ((**A**), +0.35 ± 0.98%, *n* = 3) and the C-BDDME ((**B**), −3.69 ± 8.41%, *n* = 3) exhibited stable repeatability with no significant difference between the 0-min and 120-min data points (paired, two-tailed *t*-test *p* > 0.05, ns). However, the FS-BDDME ((**C**), −20.63 ± 5.5%, *n* = 2) showed a near-significant reduction in current between the 0-min and 120-min 5-HT exposures (paired, two-tailed *t*-test *p* ≈ 0.06). (**D**–**F**) Changes in FSCV backgrounds in Tris Buffer before and after exposure to the DA waveform at 60 Hz for 24 h to simulate 6 days of constant recording. CFMEs (**D**) exhibit growth in the background, C-BDDMEs (**E**) have a very stable background with almost no change in size, and FS-BDDME (**F**) backgrounds are reduced.

The long-term stability of the devices was explored through testing the 5-HT responses over the course of 2 h. Peak oxidation currents were normalized to the 0-min data point, similar to the 5-HT fouling experiments. Both the CFME and the C-BDDME exhibit a stable 2-h response repeatability, with no significant difference found between the first and final data points (Figure 6A,B). The peak current percentage of FS-BDDME (*n* = 3) over the 120-min experiment trended downward, with a change in current of −0.0017% min^−1^*,* culminating in a final value that is approximately 80% of the original current (Figure 6C). In addition to this, 24-h etching tests at 60 Hz with the DA waveform were completed to assess material longevity and the evolution of the electrochemical background, which was assessed here to reveal potential mechanisms related to the electroactive surface area of the device and its sensitivity [10,41,53]. Figure 6D shows the evolution of the average of three CFME backgrounds after a 24-h, 60 Hz waveform application to simulate six days of constant use. The peak background current grew by 170.2 nA at the data point, which corresponds to a portion of the background near the DA and 5-HT oxidation peaks on the CFME (0.6 V on the first half of the cyclic potential waveform) (Figure 6D). As demonstrated by Takmakov et al., the application of switching potentials of >1.1 V grows the CFME background over time by creating defects as a result of etching that increase the number of adsorption sites [41]. The C-BDDME maintained a very stable background over the course of the experiment with only a very slight increase of ~3.9 nA (Figure 6E), which is to be expected given the inherent resistance of BDD to etching and wider working-potential window [27]. In alignment with the results of Figure 6C, FS-BDDMEs show a time-dependent decrease in sensitivity. Unlike the other two devices, the FS-BDDME background is reduced after 24 h (~141.3 nA reduction, Figure 6F). It is also notable that the FS-BDDMEs recordings lost signal stability during testing, resulting in an unusable signal on all devices by the end of the 24-h experiment (Figure S1). The other two electrode types remained stable.

## 4. Discussion

Neurochemical sensing can be a powerful way to improve the understanding and treatment of neurological behaviors and conditions. Using FSCV, real-time insight into neurochemical dynamics can be elucidated. However, there are limitations to chronic applications of this technique, including electrode fouling. Biofouling is considered to be the interference of any unwanted biological material that adheres to the electrode sensing surface, such as proteins, blood, soft tissue, etc. Electrochemical fouling is fouling induced by oxidative byproducts of analytes that outcompete for binding to adsorption sites on the sensing surface. This is a well-known phenomenon affecting 5-HT detection when using the standard waveform with FSCV and CFMEs [15,17,19]. In order to reduce the effects of fouling, alternative carbon materials are being explored as replacements for CFMEs. Our group has been developing an all-diamond, batch-fabricated BDDME as an alternative to the traditional CFME. As an extension of our work on biofouling [21], we tested the electrochemical fouling performance of the BDDME since it has been indicated that the two fouling types are facilitated through different mechanisms [17]. In part, we were motivated to explore this effect by our recent findings that the BDDME exhibits a more robust and consistent response to 5-HT than DA, which is practically significant due to serotonin’s roles in neuropsychiatric disease states (Figure 4D and Figure S3) [54,55,56,57].

The ultimate goal of the development of our all-diamond, freestanding BDDME is to develop a chronically implantable sensor capable of neurotransmitter detection over long periods of time. However, several challenges remain, including achieving adequate sensitivity to detect physiologically relevant concentrations of neurotransmitters in vivo, reducing fouling, and maintaining electrode stability during long-term use. The sensitivity of the BDDME is much lower than the CFME because of its ~10× smaller electroactive surface area (CFME: ~1000–1600 µm^2^, BDDME: ~100–200 µm^2^) as well as diamond’s less adsorptive material properties [28,30,41]. Femtosecond laser cutting can be used to functionalize the BDDME to a comparable sensitivity to the CFME, which has been used for numerous in vivo explorations [11,52,57,58,59,60,61]. Laser cutting converts *sp*^3^-bonded diamond to *sp*^2^-bonded graphitic content, which may come at the expense of some of the diamond’s desirable properties, including fouling resistance and background stability [27,44,62,63,64]. With the FS-BDDME, this is supported by visible inspection of a dark appearance on the electrode surface in SEM images (Figure 1F and Figure S2), as well as its similar electrochemical characteristics to that of the CFMEs (e.g., background sizes and shapes, sensitivities to DA and 5-HT, response kinetics, and LODs). Additionally, high-temperature-acid oxidation can be used to etch *sp*^2^-bonded carbon from diamonds [65,66]. We soaked BDDMEs in a 1:1:1 ratio of concentrated acids (Section 2.9). A large reduction in the FSCV backgrounds was observed with FS-BDDMEs (972.52 nA, *n* = 2) compared with a near zero (0.44 nA, *n* = 3) reduction from C-BDDMEs, indicating the removal of a larger amount of *sp*^2^-bonded carbon from the FS-BDDME (Figure S5). Post-acid cleaned FS-BDDME backgrounds were still larger than the C-BDDME backgrounds. This may be attributable to the incomplete removal of *sp*^2^-bonded carbon or an increase in the electroactive surface area due to the addition of microchannels from the FS-laser pulsing or a combination of both. It also appears that the graphitic content covers a larger span than just the conductive BDD since the darkening and laser pulses (vertical channels) can be observed across the entire face of the electrodes, as seen in the SEM images (Figure 1F and Figure S2). Given the large increase in background size and sensitivity, it is possible that laser cutting functionalizes more than just the BDD core, resulting in a substantial increase in the electroactive surface area. If so, these devices would be expected to exhibit fouling characteristics more similar to the CFME than the C-BDDME, aligning with the presented findings.

Electrochemical fouling is an important consideration in the development of a chronic 5-HT sensor since the oxidative byproducts of 5-HT are known to foul the electrode surface. The FSCV potential waveform has been adapted to avoid some effects of 5-HT fouling as demonstrated by Jackson et al. (0.2 V to 1.0 V to −0.1 V to 0.2 V at 1000 Vs^−1^ at 10 Hz) [15] and expanded upon by Dunham and Venton [14]. However, in the Dunham and Venton experiments [14], the dopamine waveform exhibits the least fouling after 25 injections of 1 µM 5-HT using CFMEs compared with the Jackson waveform and its tested variants. In this study, we observed that the C-BDDME exhibits improved electrochemical fouling performance over the CFME, with a significantly more stable peak oxidation current response to repeated injections of 5-HT. This may be explainable by low surface adsorption due to the *sp^3^*-bonded structure of BDD [29,34]. The FS-BDDME was more susceptible to 5-HT fouling than both the CFME and the C-BDDME. We observed a clear concentration-dependent fouling effect for 50 µM 5-HT compared with 5 µM 5-HT. Since 5-HT oxidation produces polymerizing radicals that form an electron-transfer-hindering film on the electrode surface [18], increased 5-HT oxidations should be expected to cause more fouling. This effect is further supported by the absence of measurable fouling to 1 µM 5-HT shown by Dunham and Venton with CFMEs [14]. The C-BDDME maintains its resistance to fouling at higher concentrations and exhibits a slower rate of fouling than the exponential decay of CFME. This reduced rate of decay could potentially reflect a contribution of a less absorptive surface of the C-BDDME [21,39,67]. The FS-BDDME fouled less than the CFME in the 50 µM experiments and had a rate of decay more closely matched to the C-BDDME than the CFME. We speculate that with large concentrations, the highly adsorptive graphitic content of FS-BDDME fouls quickly while the *sp*^3^-bonded BDD-portion retains some level of fouling resistance, leading to an “in-between” fouling performance to the CFME and C-BDDME. Optimization of the laser-cutting protocol may allow for a similar but less pronounced “in-between” effect where the FS-BDDME maintains improvements in both sensitivity over the C-BDDME and fouling resistance compared with the CFME.

Signal stability is another important goal in chronic applications where recording is required over long periods of time. The CFME is known to be very stable over short windows (~90 s) [51,52] and exhibited stable responses over a two-hour window with 5-HT in our study. However, the etching behavior of the CFME [41,68] causes a self-cleaning, surface-renewing effect and increases the number of adsorption sites on the electrode surface. In turn, this leads to increased sensitivity and a growing background current, which we observed on our 60 Hz, 24-h test. This evolution over time needs to be accounted for in the chronic setting, particularly when estimating concentrations, since there may be sensitivity changes [51,69]. Eventually, the carbon fiber will erode to an unusable level from consistent etching [31,41]. Part of the motivation for using BDD electrodes in place of the CFME is to reduce etching and maintain signal stability over longer periods of time [31]. These data confirmed that the C-BDDME had both a stable two-hour response repeatability and a very minimal background shift after the 24-h etching test. The C-BDDMEs may better maintain a stable surface without sacrificing material to etching. On the other hand, stability experiments revealed a trend toward a reduction in sensitivity over time with the FS-BDDME, likely due to the etching of the *sp*^2^-bonded carbon created by the laser, revealing the *sp*^3^-bonded diamond beneath. Thus, although the FS-BDDME greatly improves the sensitivity of the electrode in comparison to the C-BDDME, this benefit comes at the expense of stability. Additionally, we plan to investigate micromachining strategies and laser-cutting parameters to tune the level of *sp*^2^ conversion of the FS-BDDME, as FS machining has been used to form nanometer-width grooves that may provide an increased electrochemically active surface area while maintaining diamond composition [70].

As the neurochemical sensing field progresses toward improved chronic sensing capabilities, it is important to characterize emerging technologies that intend to replace traditional devices thoroughly. BDDMEs may deliver improved stability and reduced fouling but at the expense of sensitivity. Surface-modifying techniques may recover sensitivity but at the expense of stability. These trade-offs require further design iteration to leverage the benefits of the BDDME fully. However, it is also important to acknowledge that the BDDME presented here is not a one-to-one comparison with the CFME. The large disparity between the sensitivity of the BDDME and the CFME is affected by the electroactive surface area differences between the two devices [24,53]. For reference, example FSCV voltammograms from the CFME and the C-BDDME with currents normalized to estimated electroactive surface areas (CFME: 1250 µm^2^ and C-BDDME: 150 µm^2^ calculated from estimated exposed geometric surface area) are included (Figure S6). These surface-area adjusted plots result in an approximately 10× lower response from the C-BDDME. This is contrary to the 100× smaller currents from non-corrected data, indicating the sensitivity differences are largely due to differing electroactive surface areas. However, a better comparison would still include comparable sensing surface sizes and geometries. As the next step, we recommend increasing the electroactive surface area to increase sensitivity while maintaining a small device size in order to minimize glial encapsulation [71,72,73,74,75,76]. In addition to this, refinement of the BDDME through sensitivity-increasing methods, such as exploring coatings, overoxidation, waveform development, boron-doping level, and chemometric data analysis, may be viable solutions to deliver an optimized, chronic BDDME sensor with customizable, batch-fabricated architectures.

## Data Availability

Data will be made available upon request to authors.

## Supplementary Materials

The following supporting information can be downloaded at https://www.mdpi.com/article/10.3390/bios14070352/s1, Figure S1: Background-subtracted color plots for FS-BDDMEs before and after 24-h stability testing title; Figure S2: Representative SEM images of FS-BDDMEs title; Figure S3: Representative DA electrochemical fouling cyclic voltammograms. Figure S4: SEM images of electrodes pre- and post-electrochemical fouling with 5-HT. Figure S5: FSCV Backgrounds in tris aCSF before and after boiling in 1:1:1 nitric:sulfuric:perchloric acid for 30 min. Figure S6: 5-HT fouling FSCV voltammograms with current normalized to the approximate electroactive surface area of the CFME.
