# Supplementary material for: Evaluation of In Vitro Serotonin-Induced Electrochemical Fouling Performance of Boron Doped Diamond Microelectrode Using Fast-Scan Cyclic Voltammetry
Source: Biosensors (Basel). 2024 Jul 19;14(7):352. doi: 10.3390/bios14070352 (PMC11274679; doi:10.3390/bios14070352)
Supplement: Supplementary file 1 [file biosensors-14-00352-s001.zip › biosensors-3062421 Supplementary Data.pdf]

<u>Supplemental Materials</u>

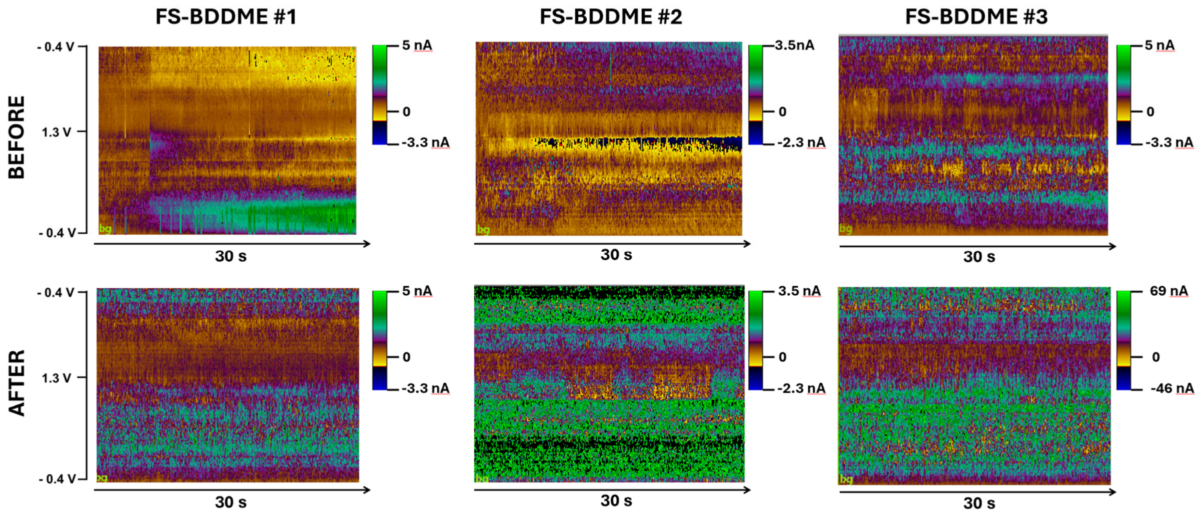

Figure S1. Background-subtracted color plots for FS-BDDMEs before and after 24-hour stability testing. These plots show the baseline noise of each individual electrode. Large increases in baseline noise can be observed in all the "After" plots as represented by the larger amounts of green and blue colors, as well as greater contrast and variability in the colors.

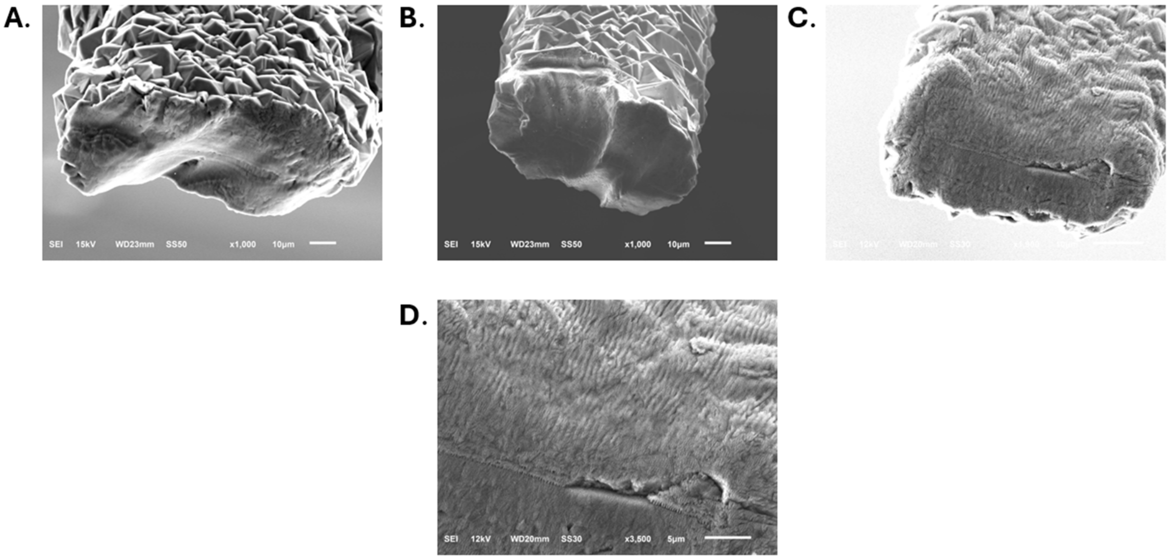

Figure S2. Representative SEM images of FS-BDDMEs. (**A**) 1000x magnification FS-BDDME. (**B**) 1000x magnification FS-BDDME (**C**) 1000x magnification FS-BDDME (**D**) 3500x magnification of the electrode shown in **C**. The darkness on the faces may indicate graphitic content. Laser pulses correspond to the striations that can be seen especially well on **C** and **D.**

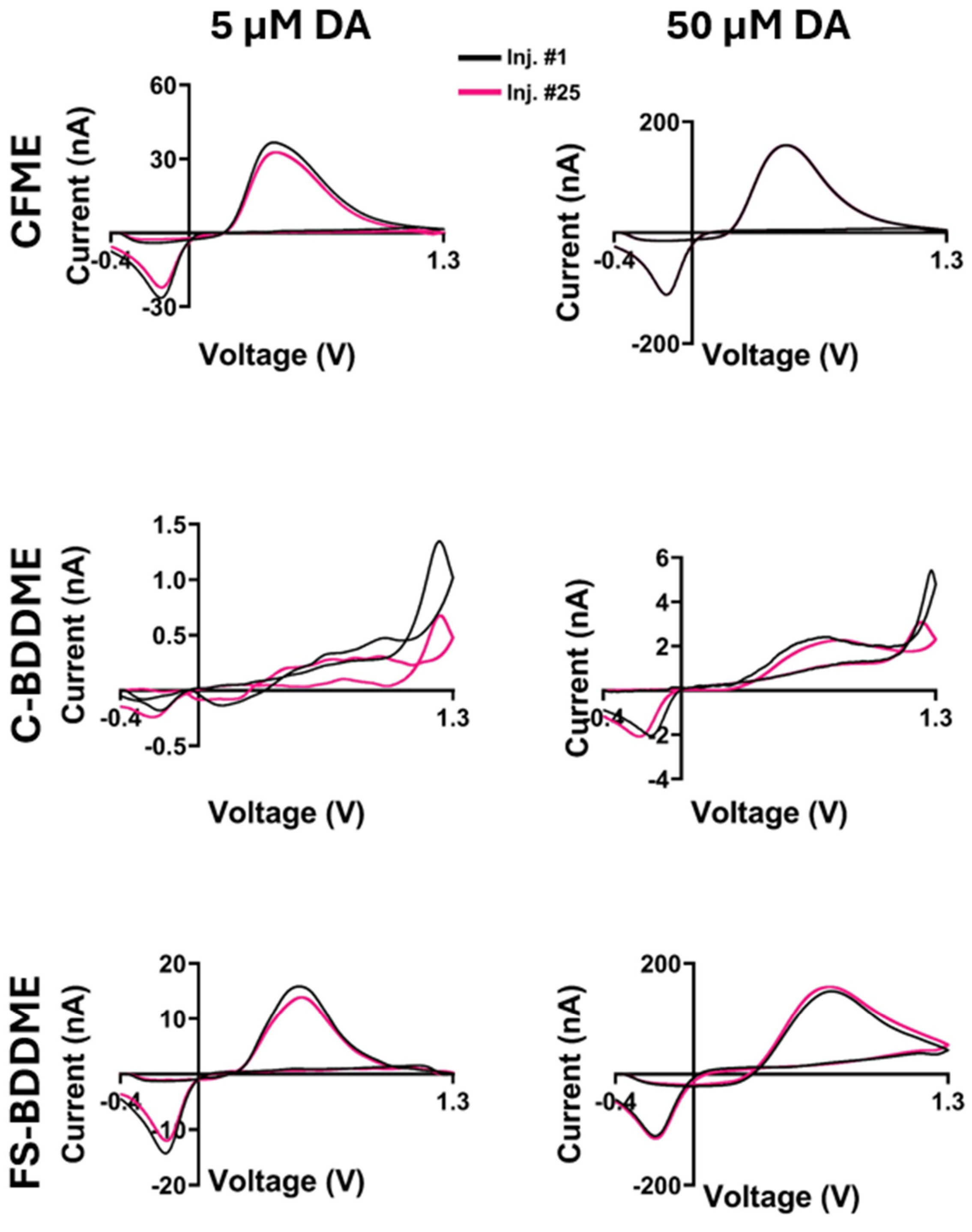

Figure S3. Representative DA electrochemical fouling FSCV voltammograms. Left column contains 5 µM DA FSCV voltammograms, right column contains 50 µM DA FSCV voltammograms. The top row is CFME, middle is C-BDDME bottom is FS-BDDME The FS-BDDME FSCV voltammograms are **not** from the same device, the others are.

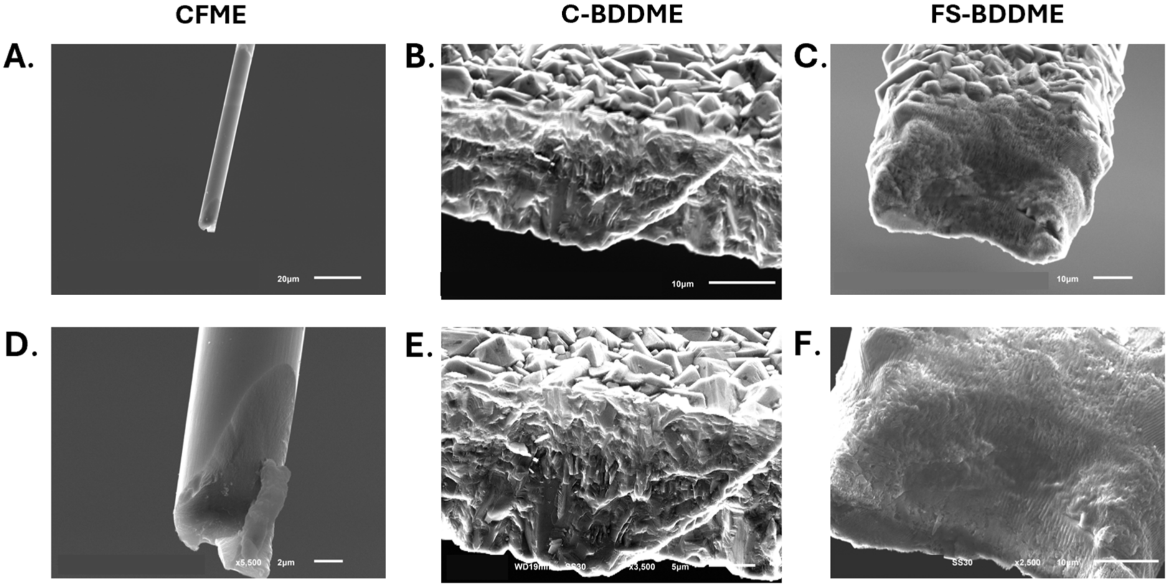

Figure S4. SEM images of electrodes pre- and post-electrochemical fouling with 5-HT. (**A-C**) Pre-fouling images of the CFME, C-BDDME, and FS-BDDME from left to right. (**D-F**) Post-fouling images.

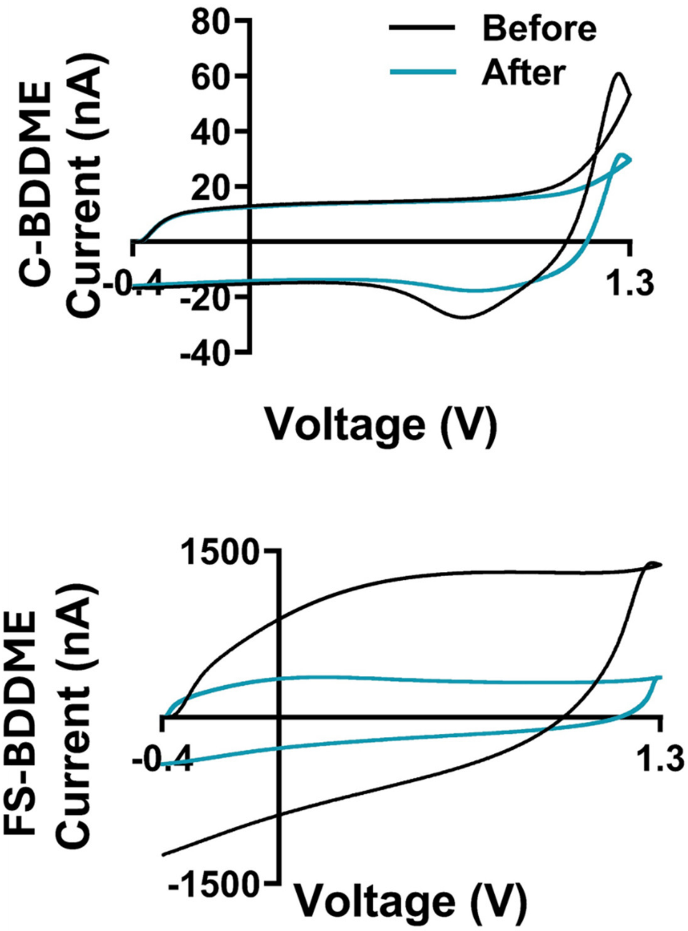

Figure S5. FSCV backgrounds in tris aCSF before and after boiling in 1:1:1 nitric:sulfuric:perchloric acid for 30 minutes. The C-BDDME background (**top)** decreased by 0.44 na measured at 0.6 V on the front scan and the FS-BDDME background (**bottom**) at 972.52 nA at the same voltage

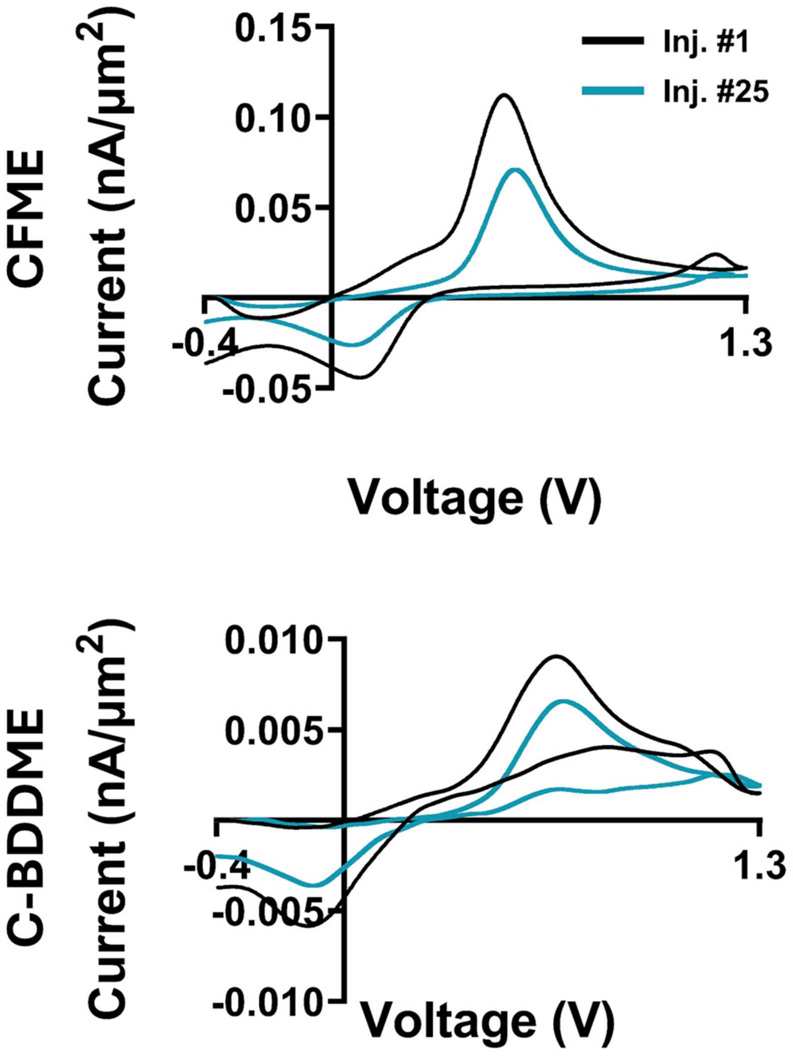

Figure S6. 5-HT fouling FSCV voltammograms with current normalized to the approximate electroactive surface area of the CFME **(top, 1250 μm²)** and the C-BDDME **(bottom, 150 μm²)**
